# Nek2-targeted ASO or siRNA pretreatment enhances anticancer drug sensitivity in triple-negative breast cancer cells

**DOI:** 10.3892/ijo.2013.1788

**Published:** 2013-01-22

**Authors:** JAEHYUNG LEE, LAUREN GOLLAHON

**Affiliations:** Department of Biological Sciences, Texas Tech University, Lubbock, TX, USA

**Keywords:** NIMA-related kinase 2, small interfering RNA, antisense oligonucleotide, paclitaxel, triple-negative breast cancer

## Abstract

Although the anticancer drugs paclitaxel and doxorubicin are commonly used to treat many solid tumors, their effectiveness is highly variable due to tumor cell resistance. Therefore, it is important to find mechanisms that can be targeted to increase the sensitivity of cancer cells to current chemotherapeutic agents. NIMA-related kinase 2 (Nek2), a serine/threonine kinase is emerging as an important oncogene because of its regulatory role in mitosis. Thus, regulation of the Nek2 expression levels may prove important as a target for cancer treatment. The purpose of our study was to determine whether drug sensitivity was increased in the triple negative breast cancer cell lines MDA-MB-231 and MDA-MB-468 by using small interfering RNA (siRNA) and antisense oligo-nucleotides (ASOs) against Nek2. To this end, MDA-MB-231 and MDA-MB-468 breast cancer cells transfected with Nek2 siRNA or ASO were exposed to various concentrations of paclitaxel and doxorubicin. Cell viability, cell cycle distribution and apoptosis were evaluated. We observed that drug susceptibility in these transfected cells was dramatically increased compared with either agent alone. FACS results showed that apoptosis was induced in siRNA- and ASO-transfected cells as expected due to the regulatory function of Nek2 in centrosome duplication. Interestingly, the cell cyle was not arrested in transfected cells. We found that siRNA and ASO against Nek2 worked synergistically with paclitaxel and doxorubicin by promoting cell apoptosis. Our results suggest that these drugs in combination with Nek2 siRNA or ASO treatment may improve the sensitivity of cancer cells during chemotherapy treatments.

## Introduction

Breast cancer is functionally classified based on molecular profiles. Estrogen receptor (ER), progesterone receptor (PR) and ErbB-2/human epidermal growth factor receptor 2 (HER-2) status are molecular markers used to determine breast cancer subtypes as well as targets for treatment (1,2). In contrast, triple-negative breast cancer (TNBC) is a breast cancer subtype defined by the lack of expression of ER, PR and HER-2. Treatment of TNBC, which often presents with a more aggressive phenotype, is more difficult due to the paucity of potential target molecules. Therefore, there is a critical need to enhance current systemic treatments and/or identify new targets for the treatment of TNBC (3–5).

Because of the strong correlation between tumor development and specific mutations in the regulatory function of certain cell cycle kinases and cyclin-dependent kinases (CDKs), their potential as targets for anticancer drug design has intensified, with the goal of overcoming the therapeutic challenges presented by TNBC (6–9). Because the centrosome cycle is regulated by protein phosphorylation and given the importance of mitotic and centrosomal kinases, they are attractive targets for anti-mitotic anticancer drugs (10–17).

NIMA-related kinase 2 (Nek2), a serine/threonine centrosomal kinase that is highly expressed and activated during the S and G2 phases, is such a target (18,19). Overexpressed Nek2 results in premature centrosome splitting, while centrosomal abnormalities, monopolar spindles and aneuploidy result from overexpression of kinase-dead Nek2 (20,21). Recently, studies have shown that Nek2 expression is elevated in various cancer cell lines, including various breast tumors (22–24). Tsunoda *et al*(23) demonstrated that Nek2 siRNA could reduce the tumor volume in mouse xenografts. However, combinational studies using Nek2 gene depletion with anticancer drugs have not been reported. In spite of the increasing evidence of the importance of Nek2 in cancer development, its role in cancer is still far from clear.

In this study, we investigated whether Nek2 depletion by antisense oligonucleotides (ASO) or small interfering RNA (siRNA) against Nek2, promoted drug sensitivity in the TNBC cell lines MDA-MB-231 and MDA-MB-468. Doxorubicin and paclitaxel treated cells were used as positive controls due to the reported problem of low vulnerability (25) with untreated cells as negative controls. Here, we show the effect of Nek2 depletion using siRNA and ASO on two different TNBC cell lines, alone and in combination with paclitaxel and doxorubicin. Alone, siRNA and ASO showed significant reductions in cell viability, mitotic spindle fiber formation and apoptosis. However, in combination with paclitaxel or doxorubicin, Nek2 depletion induced an increase in mitotic abnormalities and apoptosis above either silencing alone or anticancer drug treatment alone. Given the difficulty in effectively treating TNBC, our results suggest that either siRNA or ASO targeted against Nek2 may increase TNBC sensitivity to chemotherapy treatments.

## Materials and methods

### Cell culture and transfections

MDA-MB-231 and MDA-MB-468 breast cancer cells (ATCC, Manassas, VA) were cultured in Dulbecco’s modified Eagle’s medium (DMEM) supplemented with 10% fetal bovine serum (FBS) and penicillin/streptomycin (100 IU/ml and 100 μg/ml, respectively) under 5% CO_2_ in humid conditions at 37°C. Oligonucleotide transfections were carried out using Lipofectamine 2000 (Invitrogen, Carlsbad, CA) according to the manufacturer’s instructions. ASO and siRNA were synthesized by Integrated DNA Technologies Inc., (Coralville, IA). The antisense sequence of phosphorothioate-modified oligodeoxynucleotides against Nek2 was: 5′-GAGCCTGTGCCAATGGTG. The siRNA sequences targeted to Nek2 were 5′-CCAAGGAAAGGCAAUACUUUUdTdT-3′ (sense) and 5′-AAGUAUUGCCUUUCCUUGGUUdTdT-3′ (antisense). siRNA-A (Santa Cruz Biotechnology, Santa Cruz, CA) was introduced into cells as negative control. Null transfected cells were incubated with Lipofectamine 2000 alone. Cells were collected 24 h after transfection and analyzed for changes in transcript levels of Nek2. Cells were analyzed 48 h after transfection, by fluorescence activated cell sorting (FACS) and western blot analysis for protein expression.

### Semi quantitative reverse transcriptase PCR (RT-PCR)

Total RNA from cells was isolated using RNeasy mini kit (Qiagen, Valencia, CA) following supplier’s instructions and treated with DNase I (Promega, Madison, WI) to remove DNA contamination. To generate cDNA from purified total RNA, 1 μg of total RNA was added to the SuperScript III first-strand synthesis system reaction mixture according to the manufacturer’s (Invitrogen) protocol. Equal amounts of synthesized cDNAs were used to carry out the semi quantitative RT-PCR reactions using the GeneAmp fast PCR Master mix (Applied Biosystems, Carlsbad, CA). The Nek2 primer sequences were: forward, 5′-CCACAGACGAAGTGATGGTG-3′; reverse, 5′-TGATTTTCCCAGCGAGTTCT-3′. Glyceraldehyde-3-phosphate dehydrogenase (GAPDH) was used as control. The primer sequences were: forward, 5′-CACCACCATGGAGAAGGGTG-3′; reverse, 5′-GAGGCATTGCTGTAGATCTTGAGG-3′.

### Immunoblotting analysis

Cells were lysed with 20 mmol/l Tris-HCl (pH 8.0), 137 mmol/l NaCl, 10% glycerol, 1% Triton X-100, 2 mmol/l EDTA containing protease and phosphatase inhibitors at 4°C. A total of 20 μg of each lysate was separated by SDS-PAGE and transferred onto a nitrocellulose membrane. Mouse monoclonal antibody against Nek2 (Santa Cruz Biotechnology, 1:1,000), mouse monoclonal anti-γ-tubulin antibody (Sigma-Aldrich, St. Louis, MO, 1:10,000) and peroxidase-conjugated goat anti-mouse IgG (AnaSpec, Freemont, CA; 1:10,000) were used for immunoblot analyses.

### Cell viability assay

The XTT Cell Proliferation Kit (Roche Applied Science, Indianapolis, IN) was used to analyze cell viability. Cells were seeded at 8,000 cells/well into a 96-well culture plate in a final volume of 100 μl. The XTT mixture (50 μl) was added to the wells and incubated for 10 h at 37°C. The absorbance of the samples was measured using a Molecular Devices (Sunnyvale, CA) microplate reader at 450 nm against the reference wavelength of 650 nm. Cell viability assays were carried out at least 3 times independently.

### Cell synchronization and FACS

Cells were synchronized by double thymidine. Briefly, after 24 h incubation, 1×10^6^ cells were exposed to 2 mmole/l of thymidine for 18 h to presynchronize the cells in S phase then released by refreshing the medium for 9 h. After release, 2 mmole/l of thymidine was added to block cell cycle for 17 h followed by fresh DMEM to release cell cycle arrest allowing cells to move forward synchronously throughout G2/M. Cells were harvested 48 h later. Cell cycle distribution was analyzed by FACS. Collected cells were washed in ice-cold PBS, fixed in 70% ethanol and stored at −20°C until analysis. For FACS, DNA was stained with PBS containing 40 μg/ml of propidium iodide, 100 μg/ml of RNase A, and 0.1% Triton X-100 for 30 min at 37°C. DNA from 10,000 cells was evaluated with a FACSCalibur flow cytometer (Becton-Dickinson, Franklin Lakes, NJ) and cell cycle phases were determined using FCS Express 4.

### Immunofluorescence analysis

Cells were fixed in cold methanol and processed for immunocytochemistry. Primary antibodies: mouse monoclonal anti-γ-tubulin (Sigma-Aldrich, 1:1,000) and mouse monoclonal anti-α-tubulin (Abcam, Cambridge, MA; 1:1,000). Secondary antibodies: FITC conjugated goat anti-mouse antibody (Abcam; 1:50) and goat anti-mouse rhoda-mine conjugated antibody (Upstate, Billerica; MA, 1:500). DNA was counterstained with 4′,6-diamidino-2-phenylindole (DAPI). Cells were visualized with an Olympus IX71 inverted deconvolving epifluorescence microscope under 40X using SimplePCI software (Compix).

### Apoptosis detection

Apoptosis of treated cells was detected using FITC Annexin V/Dead cell apoptosis kit for flow cytometry (Invitrogen). After staining, fluorescence emission at 530 nm for Annexin V (FL1) and >575 nm for propidium iodide (FL3) was performed using a FACSCalibur flow cytometer (Becton-Dickinson). Data were analyzed using FCS Express 4. Each experiment was performed a minimum of three times to generate statistically relevant results.

## Results

### Expression of Nek2 following transfection with Nek2 siRNA or ASO

**Figure 1:**
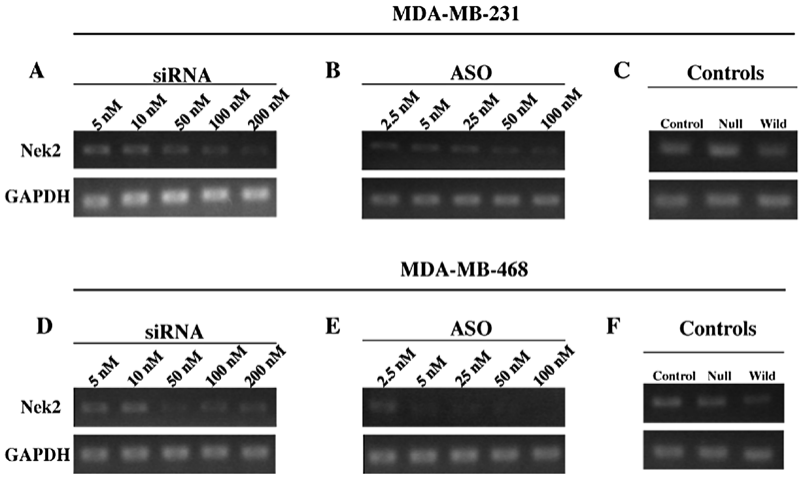
Representative semiquantitative RT-PCR of siRNA or ASO transfection against Nek2 in M231 and M468 triple negative breast cancer cells. RT-PCR analysis of M231 and M468 cells 24 h post-transfection with (A and D) 5, 10, 50, 100 or 200 nM siRNA against Nek2 and (B and E) 2.5, 5, 25, 50 and 100 nM ASO against Nek2, respectively. Each representative gel shows the Nek2 gene product (upper) and GAPDH for control (below). Transfection controls for (C) M231 and (F) M468 were analyzed using control siRNA transfection reagents, null transfection with Lipofectamine 2000 alone, and wild-type, respectively. Each experiment was conducted and analyzed from 3 independent trials.

M231 cells transfected with siRNA and ASO showed gradual loss of Nek2 mRNA expression (Fig. 1). In M468 cells, high concentrations of siRNA demonstrated significantly decreased mRNA expression (Fig. 1D). With ASO transfection, Nek2 mRNA expression declined noticeably even at 5 nM compared with 5 and 10 nM siRNA in M468 cells with higher ASO concentrations suppressing Nek2 mRNA expression very effectively (Fig. 1E).

**Figure 2:**
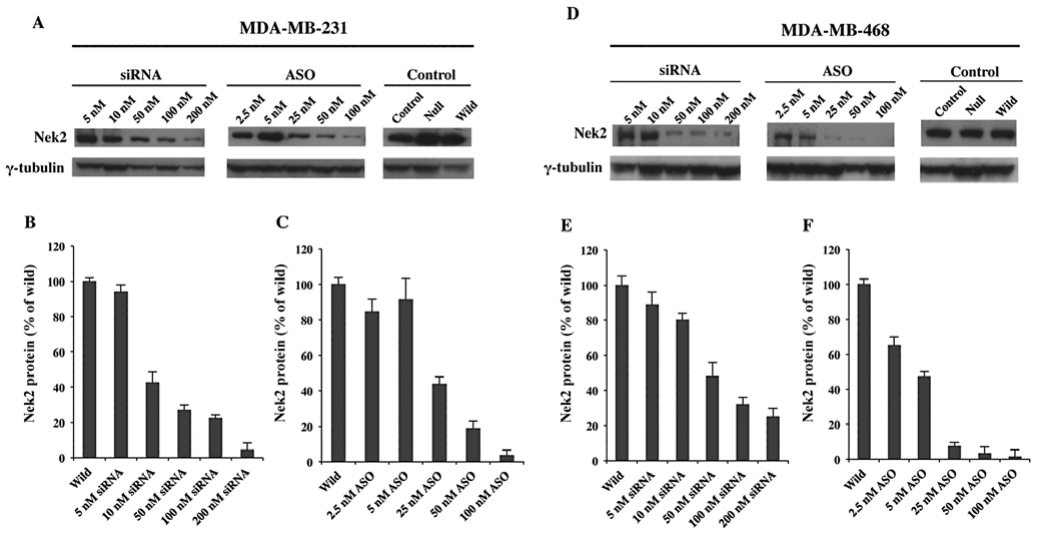
Protein expression levels of Nek2 siRNA or ASO transfected M231 and M468 TNBC cells analyzed by western blot analysis. (A and D) Nek2 protein expression after 48-h transfection in M231 and M468 cells, respectively, with 5, 10, 50, 100 and 200 nM of Nek2 siRNA (left) or 2.5, 5, 25, 50 and 100 nM of Nek2 ASO (center). Control cells were treated with control siRNA, Lipofectamine 2000 alone or untreated (right). γ-tubulin was used as the loading control for each cell line. The bar graphs represent Nek2 protein expression levels with different concentrations of (B and E) siRNA or (C and F) ASO. Nek2 protein expression is given as a percentage standardized against Nek2 expression levels in wild-type cells. Average of three independent experiments and standard deviations are shown (n=3; error bar, standard deviations).

The next step was to determine whether Nek2 depletion attenuated protein production or if expression quickly recovered. Therefore, effects of ASO or siRNA against Nek2 protein expression were analyzed 48 h post-transfection. As shown in Fig. 2, Nek2 protein expression was significantly reduced at concentrations of 50 nM siRNA (Fig. 2B). Similarly, higher concentrations of ASO (25 to 100 nM) showed a significant decrease in Nek2 expression (Fig. 2C).

M468 siRNA and ASO treatments (Fig. 2D–F) also demonstrated overall downregulation of Nek2 protein expression. Higher siRNA concentration results were not as dramatic as those observed for ASO. However, protein expression was still markedly reduced. ASO treatment in M486 cells exhibited a more profound effect when administered at higher concentrations (Fig. 2F). Analyses confirmed that Nek2 transcription and translation was successfully depleted by both siRNA and ASO and that the level of depletion was dependent upon concentration.

### Cell viability of Nek2 depleted cells

**Figure 3:**
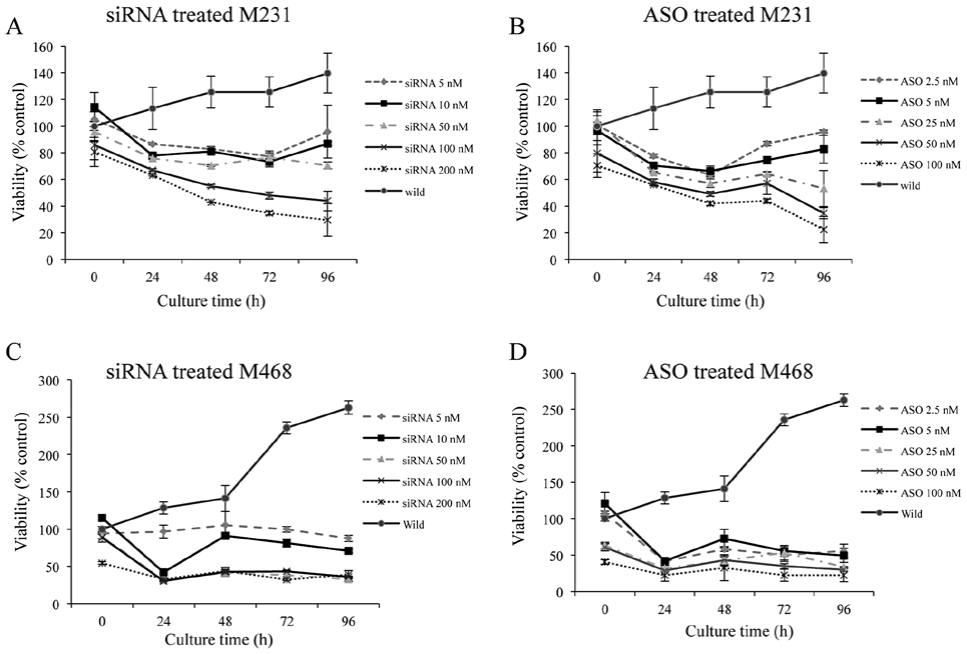
Cell viability of Nek2 siRNA or ASO transfected M231 and M468 breast cancer cells analyzed using the XTT assay. Effect of 5, 10, 50, 100 and 200 nM of Nek2 siRNA in (A) M231 and (C) M468; and 2.5, 5, 25, 50 and 100 nM of Nek2 ASO in (B) M231 and (D) M468. Percentage of cell viability is standardized against Nek2 expression levels in untreated (wild-type) cells at 0 h (n=3; error bar, standard deviations). Cells were seeded at 8,000 cells/well into a 96-well culture plate in a final volume of 100 μl. For all analyses, cell viabilities were standardized against the viability of the non-transfected cells at 0 h. Each time point indicates the time at the addition of 50 μl of XTT. The sampling measurement was recorded 10 h later. Measurements were performed in triplicate for three independent experiments.

Viability of siRNA transfected M231 cells decreased continuously from 24 to 72 h. After 96 h post-transfection, viability levels plateaued except in 5 and 10 nM siRNA transfected cells (Fig. 3A). Similarly, cell viability for ASO-transfected M231 cells demonstrated continuous decrease to 72 h with concentrations of 25 nM and greater showing no significant changes at 96 h (Fig. 3B).

Transfection of M468 cells with increasing concentrations of siRNA or ASO reduced cell viabilities more effectively (Fig. 3C and D) compared to M231 cells (Fig. 3A and B). Cell viability of M468 cells transfected with 5 nM siRNA showed no change in Nek2 expression. However, with the addition of 50–200 nM siRNA, cell viability significantly decreased after 24 h (down to 32%) and remained low (33%) through 96 h (Fig. 3C).

M468 cells transfected with Nek2-ASO demonstrated significantly reduced viabilities (between 55 to 22%) for all concentrations (Fig. 3D). Based on these results, 50 nM siRNA and 25 nM ASO were chosen as the optimal concentrations for both cell lines in the subsequent combinatorial studies of siRNA or ASO pretreatment before antitumor agent application. Cell viability of the wild-type (untreated cells) at time 0 h was normalized as 100%. Cell viability fluctuations for treated samples at time 0 h were most likely due to the effects of Nek2 depletion on cell proliferation during the 10-h XTT reaction time. Cell viability observed over 100% in the wild-type control was attributed to continued cell proliferation and metabolism through subsequent time points.

### Nek2 is crucial for mitotic spindle formation

**Figure 4:**
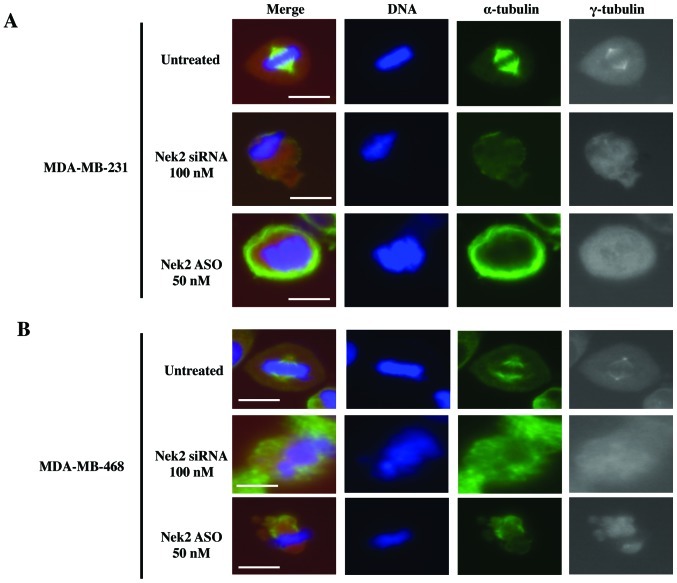
Disruption of spindles and spindle fiber formation after introduction of Nek2 siRNA or ASO. Immunofluorescence image of ASO or siRNA transfected (A) M231 and (B) M468 breast cancer cells. Both cell lines were transfected with Nek2 siRNA or ASO before cell cycle synchronization with thymidine. Synchronized cells were either untreated or transfected with Nek2 100 nM siRNA or 50 nM ASO, fixed in cold methanol, and stained with antibodies against γ-tubulin (gray image and red spot in merge) and α-tubulin (green). DNA was counterstained with DAPI (blue) (magnification; bar, 50 μm). Both Nek2 depleted cell lines demonstrated diffused spindle poles, unusual microtubule formation, weak α-tubulin staining, and misaligned chromosomes. The results are representative of 3 independent trials.

In order to determine the effects of silenced Nek2 on microtubules and spindle pole formation, immunofluorescence microscopy was used to visualize mitotic spindle structural changes. Upon release from cell cycle arrest, disrupted mitoses were observed in both siRNA and ASO transfected cells (Fig. 4A and B) whereas untreated cells exhibited no mitotic deformation. Both cell lines with silenced Nek2 demonstrated diffuse spindle poles (Fig. 4A and B, gray images) and malformed microtubules (Fig. 4A and B, green images). In addition, α-tubulin signal intensities in the spindle microtubules for transfected cells were substantially reduced compared with controls. Similar spindle structures in both Nek2 depleted cell lines were observed. These abnormal mitotic structures were classified into several types. First, Nek2 downregulated cells retained fewer microtubules (Fig. 4A and B, green images), and the γ-tubulin centrosome-associated signal was weak (Fig. 4A and B, gray images) compared to untreated cells. Lack of clear centrosomal staining against γ-tubulin suggests it is lost due to Nek2 depletion. Alternatively, duplicated centrosomes were not separated properly, or microtubule formation from the recently separated centrosomes was defective. Also, because Nek2 regulates chromosome alignment and signaling of the spindle assembly checkpoint (26), inhibition of Nek2 by siRNA or ASO lead to misaligned chromosomes at meta-phase (Fig. 4A and B, DAPI staining). This data suggests that Nek2 is required for proper mitotic spindle formation since cell death ensued as a result of abnormal microtubule generation or centrosome duplication/separation upon Nek2 silencing with either siRNA or ASO.

### The effects of Nek2 siRNA or ASO transfection on cell cycle distribution and apoptosis

Before studying combinatorial treatment efficacy for siRNA and ASO with anticancer drugs, it was important to establish whether cell cycle distribution and apoptosis levels would be changed with Nek2 depletion.

**Figure 5:**
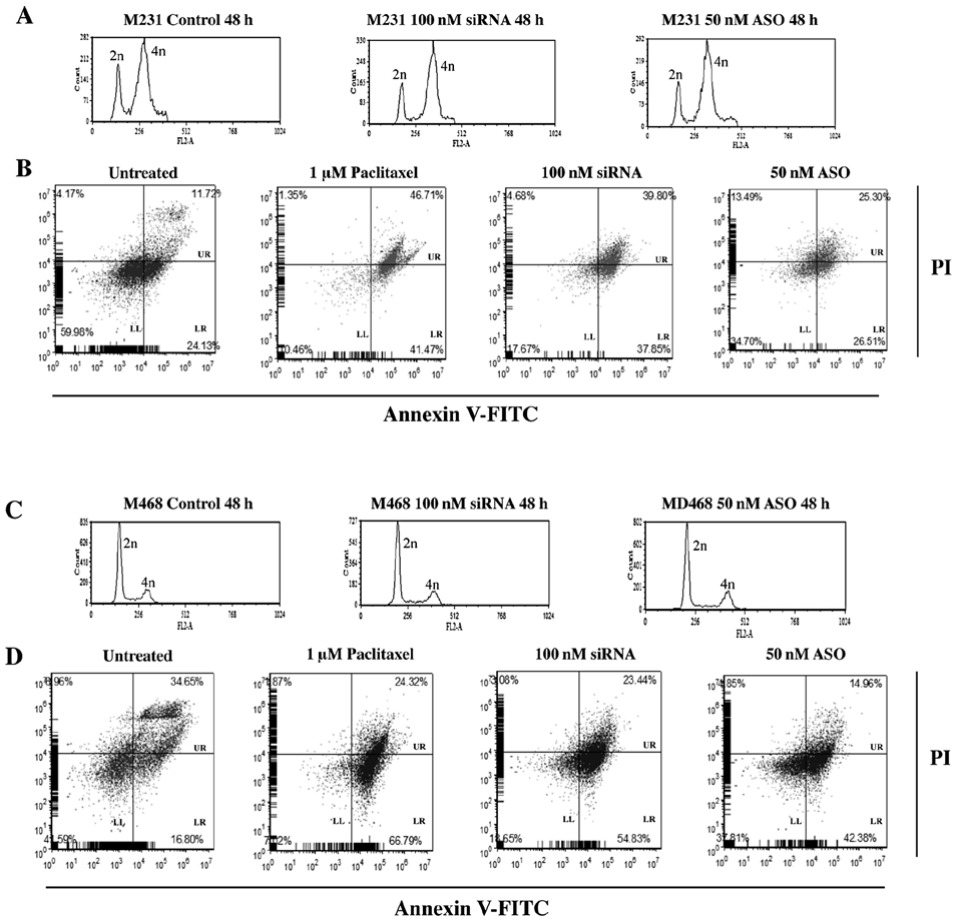
Representative results of cell cycle distribution and apoptosis for 100 nM Nek2 siRNA or 50 nM Nek2 ASO in M468 and M231 TNBC cells. (A) M231 and (C) M468 cell cycle distribution of non-transfected (left), 100 nM siRNA (middle), or 50 nM ASO (right). Cell cycle was synchronized using a double thymidine block. Time indicates released time after thymidine treatment. After cell cycle synchronization, cells were transfected with siRNA or ASO. Cells were harvested 48 h later. Cells were fixed in ethanol and the DNA content was analyzed by flow cytometry following propidium iodide (PI) staining. In A and C, the x-axis demonstrates fluorescence intensity based on DNA content, and the y-axis corresponds to the number of fluorescent cells. B and D represent parallel cell cultures stained using FITC-conjugated Annexin V and PI to analyze apoptosis using flow cytometry (LL, live cells; LR, apoptotic cells; UR, dead, necrotic and late apoptotic cells). Cells were treated with 1 μM paclitaxel, 100 nM siRNA or 50 nM ASO, respectively. Data are representative results from at least 3 independent experiments demonstrating reproducibility.

siRNA or ASO treated M231 cell lines showed little mitotic activity (Fig. 5). While a small increase in the percent of cells in 4n was observed regardless of treatment, significant changes in cell cycle profiles between control and siRNA or ASO transfected cells was not evident. Treatment with 100 nM siRNA increased cells in 4n by 10%. Cells treated with 50 nM ASO demonstrated a 6% increase in 4n compared to controls (Fig. 5A).

To determine whether Nek2 gene silencing alone caused apoptosis and how apoptosis induction compared to a commonly used anticancer drug for TNBC, cells were analyzed by FACS 24 h after siRNA/ASO transfection and paclitaxel treatment. M231 cells treated with 1 μM paclitaxel alone, showed 41% increase in cell death. Similarly, 37% increase in cell death was observed with 100 nM siRNA alone. Transfection of 50 nM ASO showed no appreciable difference in cell death (26%), over untreated cells (24%) (Fig. 5B).

M468 cell treatments demonstrated similar cell cycle distribution results. There was a 12% accumulation in 4n DNA content for control cells but only 17% for siRNA and 15% for ASO-transfected cells (Fig. 5C). However, M468 cells were more sensitive to apoptosis induction. Paclitaxel (1 μM) induced apoptosis in 66% of the treated cell population. siRNA (100 nM) increased apoptosis to 54%. Interestingly, 50 nM ASO treatment increased apoptosis to 42% compared to the 16% observed for controls.

### Combinatorial anticancer drug treatment effects on triple negative breast cancer cells

In order to address this question, paclitaxel and doxorubicin were chosen because they work through different mechanisms of action. Paclitaxel binds to the tubulin heterodimer, effectively stabilizing microtubules by inhibiting depolymerization, resulting in transient arrest in mitosis, development of a multinucleated interphase, followed by apoptosis (27,28). Doxorubicin inhibits release of DNA torsion during replication and transcription by immobilizing the topoisomerase II-DNA complex (29–31).

### The effects of combinatorial treatment on M231 cells

**Figure 6:**
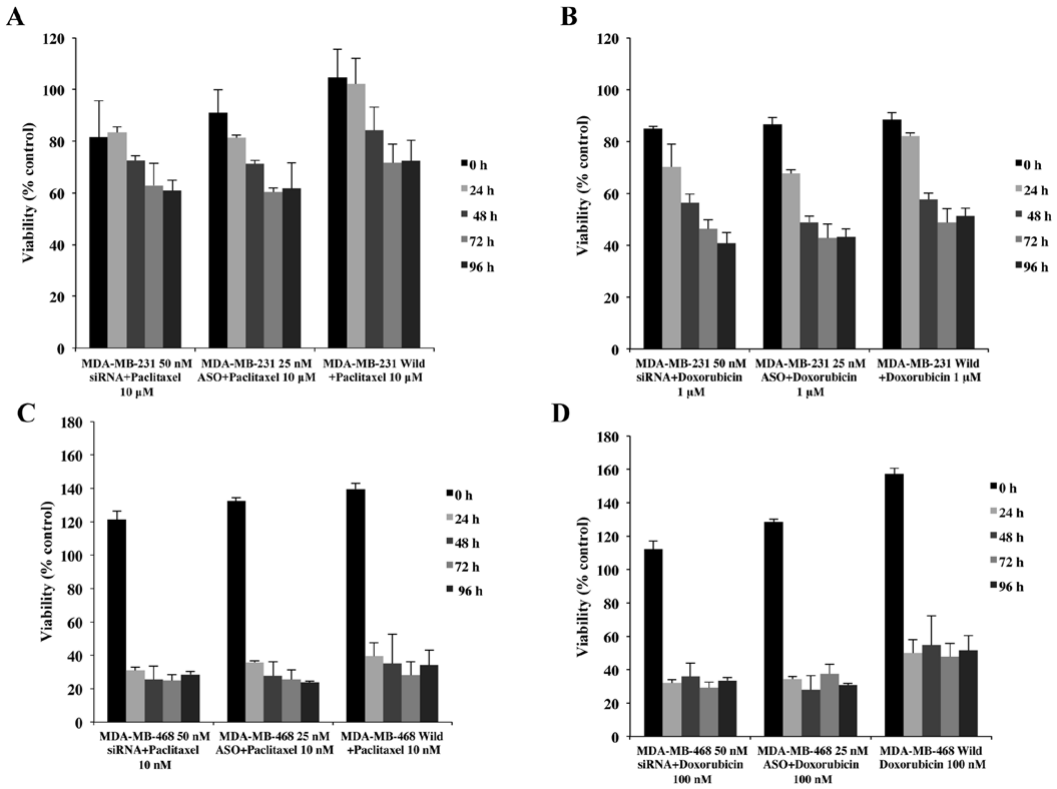
Effects of Nek2 siRNA or ASO in combination with paclitaxel or doxorubicin on the cell viability of M468 and M231 TNBC cell lines. Cell viability was determined using the XTT assay after 10-h incubation. For this study, cell viability was standardized to viability of non-transfected/untreated cells at 0 h. (A and B) Viability of 50 nM siRNA, 25 nM ASO transfected and non-transfected M231 cells with (A) 10 μM of paclitaxel treatment or (B) 1 μM of doxorubicin. (C and D) Viability of 50 nM siRNA, 25 nM ASO transfected and non-transfected M468 cells with (C) 10 nM of paclitaxel treatment or (D) 100 nM of doxorubicin. These graphs represent the results from the combinatorial study performed in triplicate from at least 3 independent experiments (error bar, standard deviations); 0 h indicates initial time point immediately after cells were treated with anticancer drug only or anticancer drug and + siRNA or ASO.

To determine whether M231 cell sensitivity to paclitaxel was augmented with this combinatorial approach, concentrations from 10 nM to 10 μM of paclitaxel were added to M231 cells pretreated with 50 nM siRNA or 25 nM ASO (Fig. 6A). Although cell viability with 50 nM siRNA + paclitaxel treated cells showed levels below paclitaxel only treated cells, there was no significant difference between siRNA combinatorial treatment and paclitaxel concentrations up to 1 μM (data not shown). However, as demonstrated in Fig. 6A, significant decreases in cell viability (60% vs. 72%) were observed in combination with 10 μM paclitaxel. Additionally, this combination of siRNA and paclitaxel generated a consistent decrease through 96 h whereas other combinations showed cell recovery at 72 or 96 h.

Interestingly, 25 nM ASO transfected M231 cells, in combination with low concentrations of paclitaxel (10, 100 nM and 1 μM paclitaxel), demonstrated decreases in cell viability from 13 to 30% (data not shown), comparable to 50 nM siRNA + 10 μM paclitaxel (Fig. 6A). Cell viability for 25 nM ASO + 10 μM paclitaxel was reduced by 19 to 29% (Fig. 6A) compared to controls. These data confirmed that Nek2 silenced by siRNA or ASO, increased M231 cell sensitivity to paclitaxel resulting in overall decreased cell viability.

Cells treated with doxorubicin in combination with siRNA or ASO against Nek2 demonstrated variable results. M231 cells treated with 10 nM doxorubicin alone showed no significant changes in cell viability until 96 h at which time, cell viability increased significantly. Cells treated with 50 nM siRNA or 25 nM ASO in combination with 10 nM doxorubicin showed significant decreased cell viability until 72 h. At 96 h cells treated with siRNA + doxorubicin recovered slightly, whereas ASO-doxorubicin mirrored the cell viability increase observed in doxorubicin alone. Similar trends were observed for combinations of siRNA or ASO with 100 nM doxorubicin.

Comparable cell viability effects were observed with 50 nM siRNA and 25 nM ASO transfected cells + 1 μM doxorubicin (Fig. 6B) and 10 μM of doxorubicin (data not shown). Cell viabilities continued to decrease through the end-point of the experiment. In summary, although 10 and 100 nM doxorubicin + siRNA or ASO appeared to increase cell viability during the culture period, the effects of 1 and 10 μM doxorubicin on M231 cells were significantly enhanced with the addition of Nek2 silencing by siRNA or ASO.

### The effects of combinatorial treatment on M468 cells

Control cells treated with 10 nM to 10 μM of paclitaxel, all showed increased viabilities at 96 h of treatment. However, even though the viability levels of transfected M468 cells were not significantly different between siRNA and ASO transfected cells with various concentrations of paclitaxel treatment, all of the cell viabilities of transfected cells were lower than non-transfected cells and remained at similar levels or showed minor increases at 96 h (Fig. 6C). In general, paclitaxel treatment in combination with 50 nM siRNA or 25 nM ASO did not significantly decrease viability compared with paclitaxel alone.

Doxorubicin treatment in combination with Nek2 inhibition had a more profound effect in M468 cells than M231 cells. No significant increase in cell viability was observed for control cells treated with 100 nM, 1 or 10 μM doxorubicin. M468 cells transfected with siRNA or ASO resulted in significantly decreased cell viability (Fig. 6D). Cells transfected with 50 nM siRNA or with 25 nM ASO plus 100 nM doxorubicin showed >34% viability. In contrast, cells in doxorubicin alone showed ∼50% viability (Fig. 6D). These results indicate that a combinatorial approach of Nek2 gene silencing with siRNA or ASO and an anticancer drug increased M468 cell sensitivity compared to anticancer drug administration alone.

Interestingly, we observed fluctuations in cell viability with 10 nM doxorubicin + 50 nM siRNA or 25 nM ASO. After 24 h, cell viability for Nek2 silenced cells + doxorubicin was 36 and 43%, respectively. In 10 nM doxorubicin treatment alone, cell viability was 53%. After 24 h cell viability increased by 20% and after 72 h an additional 40% increase was observed. Although siRNA and ASO + doxorubicin showed slight increases in cell viability, the overall increases compared to non-transfected, at 96 h, doxorubicin controls were ∼70% lower than controls.

## Discussion

In this study, we investigated Nek2 as a potential drug target in cancer treatment. Additionally, we were interested in whether pre-conditioning the cells through Nek2 silencing would augment the effectiveness of current anticancer drugs and in determining whether a combinatorial approach would maintain treatment effectiveness while decreasing anticancer drug concentrations, potentially decreasing associated side-effects. In order to address these questions, we utilized two well-characterized TNBC cell lines (MDA-MB-231 and MDA-MB-468).

When comparing the effects of Nek2 depletion by siRNA or ASO on cell viability, we observed that overall, ASO treated cells exhibited lower cell viability than siRNA treated cells. Indeed, the efficiency of siRNA and ASO is controversial. Some studies reported that when compared, siRNA was more efficient and its effect was longer than ASO (32,33). In contrast, Tsui *et al*(2005) described that the efficiency of siRNA was comparable to ASO (34). Our cell viability results suggest that the effectiveness of siRNA and ASO may be cell type dependent. Alternatively, efficiency may depend upon the target gene.

Before depleting Nek2 using siRNA and ASO, we predicted that cell cycle would be arrested in the G2/M phase based on other cell cycle kinase studies (33). Although it has been previously shown that Nek2 is one of the cell cycle kinases (18–24), our results showed that Nek2 gene silencing did not induce strong mitotic arrest at G2/M phase, but instead induced apoptosis, indicating that the role of Nek2 may be different from other cell cycle-related kinases in its regulation of cell cycle. Additionally, inactive Nek2A in human cells did not block cell cycle progression (21). It may be possible that other Nek2 family members (i.e., Nek1 to Nek11), or other cell cycle kinases such as Plk1 or Aurora A compensate for the loss of Nek2 function. However, Nek2 depleted cells have been reported to have abnormal mitotic characteristics induced through several different categories. First, Nek2 depletion interferes with centrosome duplication or maturation and arrangement of proteins including γ-tubulin, Plk1 and nucleophosmin/B23 onto the mitotic spindle poles. Secondly, Nek2 silencing induces abnormal chromosome segregation in human cells (35). Thirdly, Nek2 depletion interferes with the regulation of centrosome separation. Finally, depletion of Nek2 causes arrest of cell proliferation and increases apoptosis as a result of mitotic errors (36). As we suspected, Nek2 depletion affected the kinetochores-microtubule attachment, inducing a spindle checkpoint imbalance and abnormal clustering of kinetochore components, resulting in increased sensitivity of cells to the microtubule targeting anticancer drug paclitaxel.

The increase in cell sensitivity of Nek2-depleted TNBC cells in combination with doxorubicin may be due to the down-regulation of TRF1 and the checkpoint kinase, Chk2, inducing chromosomal abnormalities and altering the cell cycle (31). Prime *et al* demonstrated interactions between Trf1 with Mad1 and Nek2 (37). However, more study is needed to elucidate their relationship with Nek2 and the implications for cancer development.

Our results suggest that combinational administration of Nek2 depletion and these chemotherapy agents increase TNBC cell sensitivity to anticancer treatment. We observed that the effects of siRNA and ASO + anticancer agent was comparable for both cells and showed significant decreases from control non-transfected cells treated with agent alone. By pretreating the patient with siRNA or ASO therapies, the potential exists for equivalent or higher sensitization with lower dosages anticancer drugs. While achieving the same overall effect, the side-effects to the patient may be reduced.
